# Adverse clinical sequelae after skin branding: a case series

**DOI:** 10.1186/1752-1947-3-25

**Published:** 2009-01-23

**Authors:** Shahzad Raza, Khalid Mahmood, Abdul Hakeem, Sylvie Polsky, Anna Haemel, Soniya Rai, Mahadi Ali Baig

**Affiliations:** 1 Department of Oncology, New York University, NY, USA; 2 Department of Internal Medicine, Dow University of Health Science, Civil Hospital, Karachi, Pakistan; 3 Department of Cardiology, University of Cincinnati, OH, USA; 4 Massachusetts Institute of Technology, MA, USA; 5 Department of Internal Medicine, University of Wisconsin, WI, USA; 6 Gulf Medical College, Ajman, United Arab Emirates; 7 Department of Internal Medicine, Montefiore Medical Center, North Division, NY, USA

## Abstract

**Introduction:**

Branding refers to a process whereby third degree burns are inflicted on the skin with a hot iron rod or metallic object. Branding employs the phenomenon of "counter irritation," and is widely used by faith healers in developing countries for therapeutic purposes. Some methods, which are very crude and inhuman, carry a large risk of complications. The purpose of this study is to present a series of complications and to familiarize clinicians with this dangerous method of treatment.

**Case presentation:**

Four Pakistani patients, three male and one female, ranging from 25 to 60 years of age "branded" with a red hot iron rod for various medical reasons presented with severe medical complications to our tertiary care hospital. The mean duration between the procedure and presentation to the hospital was 6 days. At the time of admission, two patients had septic shock, one patient had cavernous sinus thrombosis and one patient had multiple splenic abscesses. All patients received standard care for wound management and systemic infections. Two patients eventually died during the course of treatment.

**Conclusion:**

Severe complications from branding are troublesome and the potential risks of this treatment outweigh its benefits. Globally, there is a great need for heightened awareness about the dangers of branding among patients and physicians, as this will have an important effect on patients who seek branding for various medical conditions.

## Introduction

Branding refers to a traditional practice of creating 'burns' on the skin with a hot iron rod or metallic object [1]. In several Asian and African societies where traditional medicine is still the "standard of care," branding continues to have non standard medical applications [2]. Branding employs the phenomenon of "counter irritation" which is the brief use of moderately intense pain to relieve chronic pain. A variety of methods based on this same principle have been employed in different cultures. These methods include cupping (a glass cup is heated by hot coals or flaming alcohol and inverted onto the painful area), scarification (skin over painful area is cut and allowed to bleed; this can be coupled with cupping), trepanation (scraping of the skull for headaches; producing skin abrasions on the neck for dental pain) and others [1].

There are various forms of branding which include:

• strike branding

• hypothermal (freeze) branding

• chemical branding

• electrocautery branding

• laser branding

The most common and traditional form of branding is "Strike branding" which is performed with sheet metal strips heated by a propane torch (1900 to 2100°F). The "strike" is performed by applying the heated strip to the skin. The conglomerate of heated strips forms the desired pattern on the skin post striking. Hypothermal (freeze) branding was initially introduced by cattle ranchers as an alternative method to heat branding. It involves immersing a metallic rod, similar to the metal used in strike branding, into a solution of liquid nitrogen or another cooling agent (commonly dry ice 5% in 95% pure alcohol solution). The metal sheet is then applied to the skin for a brief period. The branding leaves an indentation at the site. Sometimes caustic agents are applied directly to undamaged skin or placed on prior delineated scars from a striking or hypothermic branding. These ancient methods are crude and inhumane, causing the treatment to be more unbearable than the original complaint and carrying a large risk of complications [1-4].

Modern methods of branding as a form of body art include electrocautery branding, which uses a hot surgical cautery pen to apply the burn, and laser branding, which acts by vaporizing tissue in its path [1,3].

## Case presentation

Four patients presented to our tertiary care center with severe infectious complications. All were branded according to custom with a hot metal rod. The mean duration between branding and presentation to the hospital was 6 days. The procedure was done in a local township by faith healers as part of a cultural practice. At the time of admission, two patients presented with septic shock, one patient presented with a cavernous sinus thrombosis and another presented with multiple splenic abscesses. On examination all were clearly unwell and were found to have various medical conditions such as chronic liver disease, chronic malaria, acute glaucoma and metastatic melanoma. They were treated for the burns and wounds caused by branding and other systemic medical complications in addition to their pre-existing medical conditions. Here we discuss the outcome of these patients, including their treatment and any complications.

## Clinical cases

### Case 1

**Figure 1:**
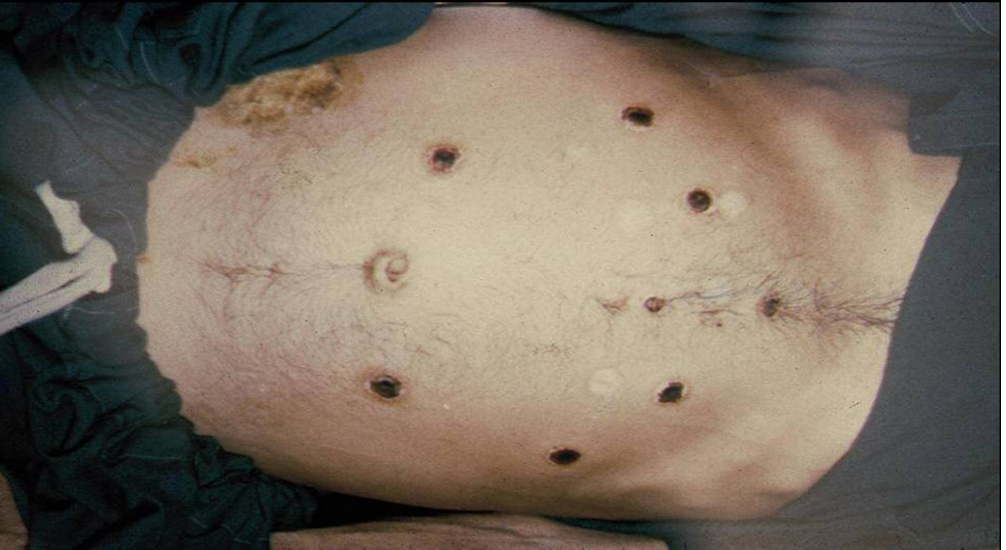
**Distributed in a circular pattern on the abdomen are round, regularly shaped symmetric necrotic ulcers with black eschar and on the right lower abdomen and extending onto the proximal thigh was a large shallow ulcer with yellow crusting but no frank purulence; all areas of skin breakdown produced by branding with a metal rod**.

A 25-year-old Pakistani male with chronic liver disease (Hepatitis C positive) underwent branding of the anterior abdomen with a heated metal rod for severe pain (Figure 1). One week later, he presented with altered mental status, high grade fever and multiple round circumscribed erythematous and indurated lesions on his abdomen ranging in size from 3 to 5 cm with black eschar and active drainage. His past medical history was noncontributory except for hepatitis C, diagnosed 10 years earlier. Ultrasound of the abdomen showed massive free fluid with splenomegaly and a shrunken liver. The ascitic fluid had a high white cell count (16,000/mm3) with 95% neutrophils. He was treated with a 3-week course of intravenous ceftriaxone and topical silver sulfadiazine and sterile dressings for 2 weeks. The fact that this was the first presentation with hepatic encephalopathy after branding, and pan sensitive *E. Coli *grew both from the branding inflicted skin wounds and ascitic fluid, points towards skin wounds inflicted by branding as the likely culprit for bacterial peritonitis and decompensated cirrhosis.

### Case 2

A 35-year-old Pakistani male with a history of chronic malaria, progressive splenomegaly and a complaint of severe left sided abdominal pain was treated by branding with a hot metal rod 7 days prior to admission. A week later he was admitted for septic shock and severe pain in the left hypochondrium. Past medical history was noncontributory except for an intermittent low-grade fever and occasional spikes of a high-grade fever with chills. On examination, he was disoriented and had multiple circular full-thickness burns to the anterior abdomen ranging in size from 2 to 5 cm across with central sparing of the skin and yellow discharge from the wounds. Blood and wound cultures grew *Staphylococcus aureus*. Abdominal ultrasound on admission showed a massively enlarged spleen with multiple rounded areas of complex echogenicity showing multiple splenic abscesses which were not seen in previous ultrasounds. He was treated with broad spectrum antibiotics and silver sulfadiazine. A splenectomy was performed and a gross examination confirmed the existence of multiple splenic abscesses which were also positive for *Staph. aureus*. Culture of pus from the spleen revealed growth of same *Staph. aureus *that were present on burn wounds complicated by skin branding. The patient died in the fourth week during the course of treatment.

### Case 3

A 60-year-old Pakistani male with a history of acute exacerbation of open angled glaucoma of the right eye underwent branding of the right temple and vertex by a faith healer. Four days later, he presented to the hospital with blurry vision in the right eye and cellulitis over the right temporal region. Further examination revealed chemosis, corneal haziness, severe restriction of ocular movements and multiple well circumscribed round full-thickness burns ranging from 2 to 5 cm with no evidence of active drainage. Fundoscopy showed papilledema and hemorrhages with engorgement of veins. On day five, similar signs appeared in the contralateral eye. His past medical history was insignificant except for glaucoma. He had no prior history of sinus headaches or ear infections. Since local infection is the major reason for cavernous sinus thrombosis (CST), the diagnosis was suspected in our patient as the typical sequelae had started after branding. Acute glaucoma can sometimes present with CST; however, he was already on treatment for open angled glaucoma and had no previous infection. The fact that he had acute exudative wounds after branding and had no prior history of ear or sinus infections points towards branding as a cause of CST. Our diagnosis was confirmed by magnetic resonance imaging (MRI). We believe this local inflammation and infection may have spread and reached one of the cavernous sinuses which may turned into an infected clot. The patient partially responded to intravenous broad-spectrum antibiotics (Amoxicillin with clavulanic acid and Ceftriaxone) and local wound management, but suffered permanent blindness.

### Case 4

**Figure 2:**
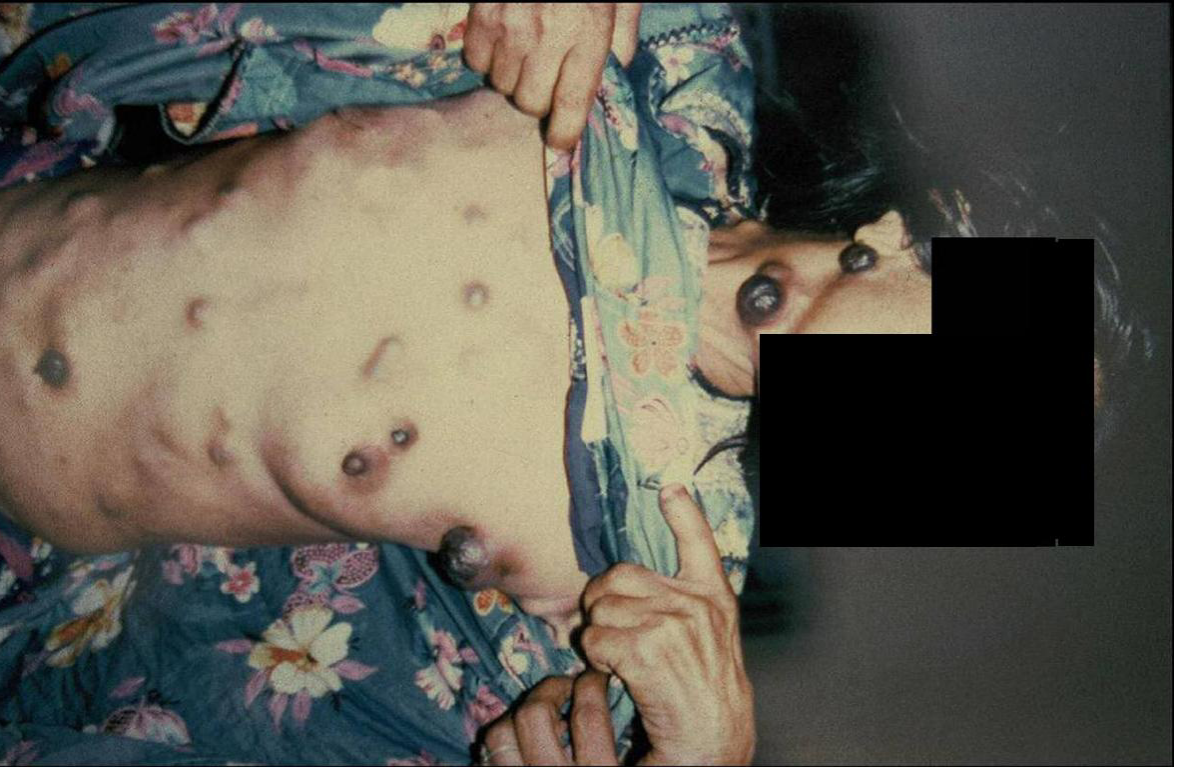
**Patient with metastatic melanoma presented with multiple areas of branding over the skin**.

A 55-year-old Pakistani female with metastatic melanoma underwent branding with a metal rod heated over coal to multiple sites of cutaneous metastases including the right ear, angle of the mandible, middle of the neck and left axillary area (Figure 2). The metallic ash was then rubbed onto the resulting wounds. Three days later she presented with fever, tachycardia and hypotension. Physical examination revealed multiple circular full-thickness burns and blisters with central sparing ranging in size from 1 to 6 cm in diameter. The burn margins were erythematous and indurated. Aggressive management of her burns was performed on an inpatient basis. Broad-spectrum oral antibiotics were administered and the wound was treated with topical silver sulfadiazine and clean dressings. Since normal skin acts as a defense mechanism in the body, in our patient, who was already immunocompromised, branding created third degree burns and breached the intact skin seeding the metastatic lesions with infection. Blood and wound cultures grew the same *Staphylococcus aureus*. She was started on intravenous vancomycin and levofloxacin. Despite aggressive measures, she died in the hospital a week later. Thus, it was either inflicting third degree burn wounds in our immunocompromised patient, or wound contamination due to unsterile techniques that had led to complications in our patient, or a combination of both.

## Results

We have presented four cases of severe complications of branding. All four patients had undergone branding for pain attributed to their chronic conditions. Mean duration between branding and admission to the hospital was 6 days. All patients presented either with acute exacerbation of their chronic conditions or new complications after branding, which were directly or indirectly associated with branding. A major complication of skin branding was local infection, which resulted in septic shock and was observed in two patients during the second week following branding, and cavernous sinus thrombosis and splenic abscesses were seen in the first and third weeks. Two patients died in hospital, one patient developed permanent unilateral blindness and one patient fully recovered during his hospital stay. Six month follow-up was available only in the one patient who recovered with no further episodes of hepatic encephalopathy, whereas the patient with unilateral blindness was lost to follow-up.

## Discussion

The study of folk medicine in ancient cultures has revealed a variety of customs and practices which have survived until the present day. One of the most common ancient techniques used for centuries to relieve pain utilizes "counter irritation" which is the application of a secondary man-made irritant to the site of the original injury. These irritants include, among others, mineral/herbal irritants, massage, firing irons and setons.

In Eastern societies where the practice of modern medicine is inaccessible to a large part of the population, patients seek branding treatments for many medical conditions such as backache, sciatica, arthritis, paralysis, facial palsy, ascites, splenomegaly, lymphadenopathy, jaundice, glaucoma, migraine headaches and sore throat [4]. Kumar S et al provided a case report of a patient with progressive weakness who underwent branding [2]. Similarly, Kaatz M et al have described body-modifying concepts and dermatologic problems of its aesthetic use [3].

The role of branding as a body art is recognized; however its role in disease management or as a subjective control of pain is unclear [1]. It is proposed that the secondary inflammatory response to the applied irritant comprising vasodilation, enzyme release, swelling, edema, blebs, vesicles and suppuration may aid leucocytes and opsonins released in the inflamed area, leading to bacterial destruction. In addition, this response aids in the rapid elimination of toxins [1]. These effects alone, however, cannot explain how counter irritation to the surface of the body can decidedly affect distant internal organs. The influence of counter-irritants may be summarized in a reflex action; for example, the production and conduction of an impulse from the periphery to nerve centers modifies the nerve function and blood supply in distant parts [5].

In our case series, faith healers used hot metal rods and coals for branding. It is hypothesized that skin breakdown and improper wound care can lead to severe infections. Thus, local wound infections resulted in exacerbation of current illness and complications like hepatic encephalopathy, subacute bacterial peritonitis, multiple splenic abscesses, cavernous sinus thrombosis and systemic bacterial sepsis in our cases. It is likely that these procedures were done in an unsterile procedure resulting in these complications and therefore carry a larger risk, which outweighs the benefits of short-term pain relief (according to popular belief but without scientific evidence). But severe medical complications of this procedure and its potential risks outweigh the benefits of short-term pain relief.

Wound management can be challenging in these patients. Patients' branded lesions typically look like multiple circular burns ranging in size from 2 to 5 cm across with central sparing of the skin [3] and they are likely to present complaining of symptoms suggestive of infection. They have diminished resistance to infection and other external noxious agents and like any other third degree burns, parenteral antibiotics fail to penetrate the dead tissue due to poor blood supply. Treatment includes local irrigation of the burns with saline solution and gentle debridement of the eschar if necessary. Infected limbs should be rested and elevated. Silver sulfadiazine or another appropriate antimicrobial agent should be applied to the wound as required [1,3]. If cellulite is present, empiric intravenous broad spectrum antibiotic coverage should be initiated (nafcillin, evofloxacin, or cephalosporin) in these patients. However, the extent of the infection and its location help determine what type and route of coverage is required.

In the long-term, branding procedures can cause disfigurement from contractures (especially over joint surfaces), scars, hair loss, keloids, orthokeratotic hyperkeratosis, acanthosis and squamous cell carcinoma (Marjolin's ulcer). Other medical complications include foreign body reaction, oral and tooth complications, aspiration and hypoxia, edema and swelling, infections and viral transmission including hepatitis and HIV [3,6-8].

Branding has recently become more fashionable in Western countries [1,3], where it is increasingly practised as body art. In these settings branding is usually performed in safe conditions, or with more precautions against serious infection, and is not used for counter irritation as it is in people whose health is already seriously compromised. Branding in Western society is described as a behavior associated with a high level of substance use, sexual intercourse and multiple partners, high risk sexual activity, smoking, marijuana use, suicidal ideation and suicidal and homicidal attempts [9-11].

There are two populations with different cultural backgrounds who seek branding for entirely different reasons. There is no direct association between the behavior associated risk problem in Western populations and the complications seen in our patients. Thus people who seek branding for body-art do not have the same sequelae of acute infections because of better sterile techniques. However, it is unclear whether the use of a sterile procedure in the chronically ill who seek branding would not still result in severe local complications. Thus a careful assessment and discussion of host factors should be an integral aspect in people who seek branding for various reasons.

## Conclusion

This article describes the complications of skin branding which still has therapeutic uses in some cultural societies. Severe medical complications of skin branding are worrisome and these risks far outweigh the benefits of these procedures. In the present study we have seen complications without therapeutic benefit, therefore universal recognition of the complications of skin branding is necessary among both patients and physicians, as it will have crucial implications in the management of patients who seek branding for a variety of medical reasons due to the lack of availability of modern medical resources.

## Consent

We hereby declare that informed consent was obtained from each subject when the patient presented to the hospital with complications of skin branding. We received permission for reporting complications, manuscript publication and photographs.

## Competing interests

The authors declare that they have no competing interests.

## Authors' contributions

SH contributed to analysis of the data, creation of the conceptual design, literature search, interpretation of results, and writing of the manuscript. MB also aided in interpreting the results, searching the literature, acquiring data, and writing the manuscript. KM provided the background and design of the study and a careful review of the manuscript. SP revised and edited the final manuscript, SR and AHD helped with the design of the study, and searched the literature. AH provided background on the study, aided in acquisition of data, and helped with the design and interpretation.
